# The Hidden Spectrum Within Eating Disorders: Clustering Neurodivergent Traits and Sensory Sensitivity

**DOI:** 10.1002/eat.24529

**Published:** 2025-08-17

**Authors:** Paolo Meneguzzo, Marta Magno, Alice Garolla, Elisa Bonello, Elena Tenconi, Patrizia Todisco

**Affiliations:** ^1^ Department of Neuroscience University of Padova Padova Italy; ^2^ Padova Neuroscience Center University of Padova Padova Italy; ^3^ Eating Disorder Unit, Casa di Cura “Villa Margherita”—Neomesia Arcugnano Italy; ^4^ Unit of Epidemiological Psychiatry and Digital Mental Health IRCCS Centro San Giovanni di Dio Fatebenefratelli Brescia Italy; ^5^ Psycho‐Nutritional Center Verona & Vicenza Italy

**Keywords:** cluster analysis, eating disorders, neurodiversity, personalized intervention, sensory sensitivity, social cognitionautistic traits

## Abstract

**Objective:**

Emerging research suggests that autistic traits and sensory sensitivities are prevalent among individuals with eating disorders (EDs), particularly females. Traditional diagnostic approaches may overlook the heterogeneity of neurodevelopmental features within this population. A person‐centered approach could uncover meaningful subgroups and guide individualized treatment strategies. This study aimed to identify neurocognitive profiles among cisgender female ED patients, focusing on autistic traits, sensory sensitivity, social cognition, and clinical severity, through a dimensional and person‐centered clustering approach.

**Method:**

A total of 164 cisgender female patients were assessed using the Autism Spectrum Quotient (AQ‐10), Girls Questionnaire for Autism Spectrum Condition (GQ‐ASC), Sensory Perception Quotient (SPQ‐10), Story‐based Empathy Task (SET), and clinical measures including the Eating Disorder Examination Questionnaire (EDE‐Q), Body Uneasiness Test (BUT), and Symptom Checklist‐90‐Revised (SCL‐90‐R). Hierarchical cluster analysis (Ward's method) was conducted on standardized scores. Between‐cluster comparisons and multinomial logistic regression assessed the robustness and predictive validity of the cluster solution.

**Results:**

Four distinct clusters emerged, differing significantly in autistic traits, sensory sensitivity, social cognition, and ED severity (all *p* < 0.001). Profiles included a neurodivergent high‐risk group, a cognitively compensated group, a sensory‐reactive group, and a classically symptomatic group. Multinomial logistic regression predicted cluster membership with 94% accuracy.

**Discussion:**

Neurodevelopmental dimensions meaningfully differentiate ED subgroups and may inform more personalized, stratified care. These findings highlight the importance of integrating autistic traits, sensory processing, and social cognition into ED assessment and treatment planning.

**Public Significance Statement:**

This study identifies four distinct neurodevelopmental and clinical profiles among women with eating disorders, defined by differences in autistic traits, sensory sensitivity, and social cognition. Understanding these profiles can help clinicians recognize the diversity of presentations in eating disorders and adapt interventions to the specific needs of each individual, particularly those with neurodivergent features, ultimately promoting more personalized and effective care.

**Summary**

- Autistic traits and sensory sensitivities are increasingly recognized in individuals with eating disorders, especially females, but are often under assessed using standard tools.
- This study used a dimensional and person‐centered clustering approach to identify four distinct neurodevelopmental and clinical profiles in 164 cisgender female ED patients.
- The results highlight the presence of a high‐risk neurodivergent group characterized by high levels of autistic traits, sensory sensitivity, impaired social cognition, and high clinical severity.
- Gender‐sensitive tools such as the GQ‐ASC were more effective than traditional measures (AQ‐10) in detecting autism‐related traits relevant to ED presentation and treatment planning.
- These findings support a move toward personalized, neurodiversity‐informed approaches in the assessment and care of eating disorder patients.

## 1 Introduction

Over the past decade, growing clinical and scientific attention has focused on the intriguing overlap between eating disorders (EDs) and autism, reflecting a broader trend toward understanding psychiatric disorders as interrelated rather than discrete entities (Carpita et al. 2022). Once considered unrelated conditions—one rooted in sociocultural pressures and body image ideals, the other in early‐onset neurodevelopmental differences—recent evidence suggests that they may share more in common than previously thought. Across diagnostic categories such as anorexia nervosa (AN), bulimia nervosa (BN), binge eating disorder (BED), and other specified feeding or eating disorders (OSFED), a significant proportion of patients exhibit high levels of autistic traits (Boltri and Sapuppo 2021; Dell'Osso et al. 2018; Saure et al. 2020). These include cognitive rigidity, extreme detail‐focused thinking style, difficulties in social cognition, sensory sensitivities, and atypical emotional processing—all of which may influence how individuals experience, develop, and respond to ED symptomatology (Kerr‐Gaffney et al. 2019; Kinnaird, Norton, Stewart, and Tchanturia 2019; Westwood et al. 2016). These features resonate strongly with core ED behaviors—such as rigid dietary rules, ritualized eating patterns, social withdrawal, and heightened sensory sensitivity to body‐related stimuli or food textures—highlighting a potential shared neurocognitive vulnerability that may benefit from dimensional approaches and precision psychiatry perspectives (Ghiotto et al. 2022; Kerr‐Gaffney, Halls, et al. 2020; Nickel et al. 2019).

Recent years have seen growing recognition of the intersection between autism and EDs, particularly in individuals with AN restricting subtype (Saure et al. 2021; Warrier et al. 2018). Several studies have reported that individuals with AN‐R tend to score higher on measures of autism‐related traits compared to those with other ED diagnoses, possibly due to overlapping features such as cognitive rigidity, social difficulties, and sensory sensitivities. However, while AN has traditionally been the focus of this line of research, there is increasing evidence that autism‐related traits are not limited to this group. Contemporary findings suggest that traits such as difficulties with social communication, inflexible thinking, and atypical sensory experiences may be present across a broader spectrum of ED presentations, including BN and BED (Dell'Osso et al. 2018; Makin et al. 2025; Westwood et al. 2017). For instance, neurodevelopmental traits such as impulsivity, attentional dysregulation, and heightened sensory reactivity have been observed in individuals with BN and BED. Neuroimaging and theoretical models further support shared mechanisms across ED subtypes, implicating altered interoceptive awareness, social cognition, and sensory integration in both autism and EDs (Adams et al. 2024; Brown and Stokes 2020). These traits are not merely co‐occurring features but may represent transdiagnostic neurodevelopmental dimensions that shape the onset, maintenance, and clinical expression of EDs (Ingrosso et al. 2024). This shift from a categorical to a dimensional perspective supports the integration of neurodiversity‐informed frameworks in ED assessment and treatment.

Recent neuroimaging research has revealed overlapping alterations in brain regions associated with social cognition, interoception, and emotional regulation in both people with autism and AN. Structural differences in the medial prefrontal cortex, temporoparietal junction, and insula—areas involved in theory of mind and bodily awareness—have been observed across both conditions (Halls et al. 2022). However, a recent study found no direct association between autistic traits and cortical morphology in individuals with acute or weight‐restored AN, suggesting that these traits may not reflect shared neuroanatomy but instead arise from distinct functional or developmental pathways (Sader et al. 2025). Similar patterns of neural alteration have also been identified in other EDs, such as BN and BED, particularly in frontostriatal and limbic areas (e.g., anterior cingulate cortex, insula, and orbitofrontal cortex) that are involved in emotional regulation, reward sensitivity, and interoceptive awareness (Donnelly et al. 2018; Frank 2013). These findings underscore the complexity of neurodevelopmental contributions to EDs and support the need for dimensional, transdiagnostic approaches.

One emerging domain of interest is sensory sensitivity, now recognized as a core diagnostic feature of autism and increasingly described in ED populations (Ingrosso et al. 2024). ED patients often report heightened sensitivity to textures, sounds, bodily sensations, or environmental stimuli—features that may contribute to avoidant eating, distress during meals, and altered body perception (Kinnaird, Norton, Pimblett, et al. 2019; Nimbley et al. 2022; Petitpierre et al. 2021). Despite this, the interaction between autistic traits and sensory profiles remains underexplored, particularly in how these traits cluster or differentiate within ED populations.

Another layer of complexity is the historical underrecognition of autism in females (Bargiela et al. 2016). Diagnostic criteria and screening tools have historically been developed based on male presentations, contributing to systematic underdiagnosis and delayed recognition in autistic women (Lockwood Estrin et al. 2021). Many autistic women, including those with EDs, use camouflaging strategies—efforts to mask or compensate for social difficulties—which may obscure core neurodevelopmental features and confound clinical evaluation (Dell'osso et al. 2021; Hull et al. 2020). This diagnostic bias has important clinical implications, as undetected autistic traits in ED patients are associated with poorer treatment outcomes and higher psychological distress. Addressing this gap requires the use of gender‐sensitive assessment tools and a more inclusive, neurodiversity‐affirming framework that acknowledges variation in neurocognitive functioning as part of the broader psychiatric landscape.

Social cognition, broadly defined as the capacity to understand and respond to the mental and emotional states of others, has garnered increasing attention in the context of EDs. Difficulties in theory of mind, emotion recognition, and empathy have been reported across various ED subtypes, particularly in AN, and are often associated with higher symptom severity, interpersonal difficulties, and poorer treatment response (Caglar‐Nazali et al. 2014; Meneguzzo et al. 2020; Oldershaw et al. 2018, 2019). These impairments may contribute to social withdrawal, reduced emotional attunement, and heightened self‐focused attention—features that can exacerbate body image concerns and disordered eating behaviors (Duarte et al. 2016; Meneguzzo et al. 2024). Moreover, deficits in social cognition overlap with those seen in autism spectrum conditions, further complicating the clinical presentation of patients with both EDs and neurodevelopmental traits (Kerr‐Gaffney, Halls, et al. 2020). Although similar behavioral difficulties may emerge, the underlying mechanisms may differ (Kerr‐Gaffney, Halls, et al. 2020; Kerr‐Gaffney, Mason, et al. 2020). In individuals with EDs alone, social cognitive impairments may be linked to emotional dysregulation or hypermentalizing, whereas in those with overlapping neurodivergence, they may reflect reduced spontaneous mentalizing, atypical social motivation, or the use of compensatory strategies such as masking. Given its potential role in both the onset and maintenance of EDs, social cognition represents a key transdiagnostic dimension warranting further investigation.

Given these considerations, the present study aims to investigate the distribution and interplay of autistic traits and sensory sensitivities in a clinical sample of cisgender female patients with EDs, adopting a dimensional and person‐centered approach aligned with the principles of precision medicine. This involves tailoring clinical understanding and treatment strategies to individual differences in neurocognitive functioning, sensory processing, and social cognition. By applying hierarchical cluster analysis, we move beyond categorical classification to identify latent psychological profiles that may better capture the heterogeneity observed in EDs. This clustering approach is exploratory and data‐driven, conducted without predefined hypotheses about the number or structure of resulting profiles. Uncovering subgroups with distinct clinical and neurodevelopmental patterns may support a more nuanced and individualized understanding of EDs and contribute to the development of personalized, neurodiversity‐informed interventions.

## 2 Methods

### 2.1 Participants

The sample consisted of 164 cisgender female inpatients consecutively referred to the EDs Unit at the Casa di Cura Villa Margherita‐KOS group (Arcugnano, Vicenza, Italy). All participants were White and of Italian nationality. Participants were enrolled as part of a broader clinical and research protocol focused on the clinical evaluation of the specific treatment approach. These features may influence patients' engagement with standard inpatient treatment for EDs, which in our setting is based on cognitive‐behavioral therapy and includes personalized third‐wave interventions, such as mindfulness, emotion regulation, and schema‐based strategies (Todisco et al. 2020, 2023). Of the total sample, 34 participants (20.7%) were adolescents (aged 16–17 years), and 130 were adults (≥ 18 years).

Inclusion criteria were: (a) a diagnosis of an ED according to DSM‐5‐TR criteria (American Psychiatric Association 2013), including AN restrictive (ANr) or binge‐purge subtype (ANbp), BN, BED, or OSFED; and (b) the ability to understand and complete the study questionnaires with or without assistance. Exclusion criteria included intellectual disability or current psychotic disorder. Participants aged 16 years and older were included in the study, consistent with the minimum age required for admission to the adolescent unit of the clinic. ED diagnoses were assigned by licensed clinicians at the inpatient center based on DSM‐5 criteria, following standard multidisciplinary assessment procedures. All participants were assessed at the start of their inpatient treatment, before beginning structured psychological interventions within the facility. According to national guidelines in the Italian healthcare system, inpatient admission is only granted after at least one prior outpatient treatment has failed, ensuring a history of treatment resistance across the sample.

Data were collected from patients consecutively admitted to the inpatient unit between fall 2022 and spring 2024. Inclusion criteria closely matched the unit's standard admission criteria; only two patients were excluded due to male sex and seven due to missing data. The inpatient unit primarily admits individuals with AN, BN, and OSFED. Patients with Avoidant/Restrictive Food Intake Disorder (ARFID) are not routinely referred to this treatment setting and are typically managed in specialized outpatient or pediatric pathways; as a result, no participants with a diagnosis of ARFID were included in the present sample.

The sample included both adult and underage participants. The questionnaires were administered by trained clinicians and reviewed for completeness and accuracy. Informed consent was obtained from all adult participants. For minors, consent was obtained from a parent or legal guardian, and assent was obtained from the minor. The study protocol was approved by the local Ethics Committee and was conducted in accordance with the Declaration of Helsinki and relevant Italian regulations regarding research involving minors.

### 2.2 Measures

#### 2.2.1 Clinical and Anthropometric Data

Participants' body weight (kg) and height (m) were measured at admission to calculate the Body Mass Index (BMI; kg/m^2^). A psychiatrist specialized in ED evaluated all participants to confirm eligibility according to inclusion and exclusion criteria.

#### 2.2.2 Assessment of Autistic Traits

Autistic traits were evaluated using two complementary measures to enhance sensitivity, particularly relevant given the historical underdiagnosis in females. The Autism Spectrum Quotient—10 items (AQ‐10) is a validated brief screening instrument assessing core autistic characteristics such as social communication difficulties, attention to detail, and cognitive rigidity, with higher scores indicating greater autistic traits (scores ≥ 6 suggesting clinical significance) (Booth et al. 2013; Ruta et al. 2012). The scale showed acceptable internal consistency in the current sample (Cronbach's *α* = 0.76). Additionally, the Girls Questionnaire for Autism Spectrum Conditions (GQ‐ASC) provided a gender‐sensitive assessment of traits frequently observed in females, such as social camouflaging behaviors, heightened sensory sensitivities, restricted imaginative play, and narrow interests (scores ≥ 54 indicating clinically significant traits) (Brown et al. 2020). Although originally developed for adult women, the GQ‐ASC has been successfully used in adolescent populations (Rynkiewicz and Lucka 2018), including girls aged 12–18 years. Given the early‐onset nature of ED and the gendered presentation of neurodivergent traits, we deemed the GQ‐ASC appropriate for both adolescent and adult participants in our sample. The combined use of these tools allowed a comprehensive evaluation tailored to female presentations of autism spectrum traits. As there is currently no validated Italian version of the GQ‐ASC, we translated the instrument from English using a standard forward–backward translation procedure. Two bilingual researchers independently translated the items into Italian, followed by a back‐translation by a third bilingual expert. Discrepancies were resolved through discussion to ensure conceptual equivalence. In the present sample, the total score demonstrated good internal consistency (Cronbach's *α* = 0.81), with subscale alphas ranging from 0.71 to 0.78.

#### 2.2.3 Sensory Sensitivity

Individual differences in sensory processing, including hypersensitivity across visual, auditory, tactile, and interoceptive modalities, were assessed using the Sensory Perception Quotient—10 items (SPQ‐10), with lower scores indicating greater sensory hypersensitivity (Tarantino et al. 2024; Tavassoli et al. 2014). Although it has been employed in previous studies, its psychometric validation is limited compared to the original version, and findings should be interpreted with this in mind. The scale showed good internal consistency in the current sample (Cronbach's *α* = 0.73).

#### 2.2.4 ED Psychopathology and Body Image Disturbance

Eating‐related psychopathology was measured using the Eating Disorder Examination Questionnaire (EDE‐Q), a validated self‐report instrument assessing dietary restraint, eating concerns, and shape and weight concerns, providing a global severity score (Calugi et al. 2017; Fairburn and Beglin 1994). The global scale showed good internal consistency in the current sample (Cronbach's *α* = 0.85). To evaluate body image disturbances, participants completed the Body Uneasiness Test (BUT), which measures body dissatisfaction and related psychopathological features, including avoidance behaviors (A), body image concerns (BIC), compulsive self‐monitoring (CSM), weight phobia (WP), and depersonalization (D) (Cuzzolaro et al. 2006). The global severity index showed good internal consistency in the current sample (Cronbach's *α* = 0.82).

#### 2.2.5 General Psychopathology

Overall psychological distress and symptom dimensions were assessed using the Symptom Checklist‐90‐Revised (SCL‐90‐R) (Derogatis and Lazarus 1994; Prunas et al. 2012). This validated instrument yields a Global Severity Index (GSI) and nine specific subscale scores (somatization, obsessive‐compulsiveness, interpersonal sensitivity, depression, anxiety, hostility, phobic anxiety, paranoid ideation, and psychoticism), with higher scores indicating greater distress. The GSI showed good internal consistency in the current sample (Cronbach's *α* = 0.84).

#### 2.2.6 Social Cognition and Theory of Mind

Participants also completed the computerized Story‐based Empathy Task (SET) (Dodich et al. 2015), which comprises 18 illustrated comic‐strip‐style narratives designed to evaluate core dimensions of social cognition. The task includes three subscales: Causal Inference (CI), which assesses understanding of cause‐effect relationships; Emotional Attribution (EA), the ability to recognize others' emotional states; and intention attribution (IA), which measures the capacity to infer others' intentions or goals. Each subscale consists of 6 items, and responses are scored as either correct (1) or incorrect (0), resulting in a maximum of 6 points per subscale and a total score ranging from 0 to 18. Higher scores indicate better Theory of Mind functioning. The SET has shown good psychometric properties in both clinical and normative Italian populations.

#### 2.2.7 Data Analysis

Data analysis was performed using JASP (version 0.19.3), with additional visualizations created using Python (version 3.11). Descriptive statistics were computed for all demographic and clinical variables. Pearson correlations were conducted to examine associations between measures of autistic traits (AQ‐10, GQ‐ASC), sensory sensitivity (SPQ‐10), and social cognition (SET subscales: CI, EA, IA). Clinical cut‐offs for AQ‐10 (≥ 6) and GQ‐ASC (≥ 54) were applied, and the concordance between these instruments in classifying individuals with clinically significant autistic traits was evaluated by analyzing their overlap.

To identify latent psychological profiles within the sample, we conducted a hierarchical cluster analysis using Ward's method with Euclidean distance—an agglomerative clustering technique appropriate for continuous psychological variables—applied to standardized scores (*z*‐scores) of key neurodevelopmental and clinical measures (AQ‐10, GQ‐ASC total score, SPQ‐10, SET subscales, and EDE‐Q global score). The optimal number of clusters was determined through visual inspection of the dendrogram, which clearly supported a four‐cluster solution based on interpretability and clinical differentiation.

Subsequent post hoc analyses characterized each identified cluster in terms of diagnostic composition, sensory profiles, social cognitive functioning, ED severity (EDE‐Q), body image disturbance (BUT total score), and general psychopathology (SCL‐90‐R). A radar plot visualizing standardized psychological profiles allowed intuitive comparison across clusters. Given that assumptions of normality and homogeneity of variances may be violated in clinical populations, between‐cluster differences were assessed using both parametric (one‐way ANOVA) and non‐parametric (Kruskal–Wallis) tests to ensure robust and consistent findings irrespective of distributional assumptions.

Finally, to assess the predictive validity and clinical utility of the identified clusters, we performed a multinomial logistic regression using standardized scores of autistic traits, sensory sensitivity, social cognition, ED severity, body image disturbance, and global psychopathology to predict cluster membership. Model performance was rigorously evaluated on a held‐out test set; reporting standard metrics such as precision, recall, and F1‐score.

Age was not used as an input variable in the clustering analysis but was examined post hoc across the resulting clusters to assess potential confounding effects.

## 3 Results

The final sample consisted of 164 cisgender female participants diagnosed with EDs. Diagnostic distribution was as follows: 45 individuals with ANr, 20 with ANbp, 42 with BN, 35 with BED, and 22 with OSFED. All participants were admitted due to severe eating psychopathology, substantial functional impairment, elevated psychological distress, or relevant medical complications, justifying inpatient treatment.

Participant characteristics by diagnostic group, including age, BMI, and illness duration, are presented in Table 1.

**TABLE 1:** Descriptive statistics of demographic and clinical variables by diagnostic category.

Diagnosis	*N*	Age	BMI	Illness duration
ANr	45	22.76 (7.47)	15.33 (1.66)	4.09 (3.59)
ANbp	20	24.95 (8.96)	16.39 (1.56)	8.45 (8.26)
BN	42	22.05 (4.48)	22.71 (3.04)	6.14 (3.85)
BED	35	32.69 (13.94)	40.51 (8.41)	12.26 (10.52)
OSFED	22	23.91 (10.80)	21.17 (2.38)	5.00 (5.46)

*Note*: Values are reported as means and standard deviations (SD).

Abbreviations: ANbp, anorexia nervosa binge‐purging subtype; ANr, anorexia nervosa restricting subtype; BED, binge eating disorder; BMI, body mass index; BN, bulimia nervosa; OSFED, other specified feeding or eating disorders.

### 3.1 Psychopathology

Participants demonstrated marked ED symptomatology, with mean global scores on the EDE‐Q (*M* = 4.27, SD = 1.07) exceeding established clinical thresholds (≥ 2.3–2.8), indicating significant clinical impairment (Calugi et al. 2017). High severity was consistently observed across all subscales: restraint (*M* = 3.45, SD = 1.73), eating concern (*M* = 3.65, SD = 1.51), shape concern (*M* = 5.13, SD = 1.09), and weight concern (*M* = 4.61, SD = 1.33).

Body image disturbances assessed by the BUT also revealed clinically significant distress, with the total score (*M* = 3.18, SD = 0.90) above the clinical cut‐off (≥ 1.2). High scores were observed for WP (*M* = 3.66, SD = 1.03), BIC (*M* = 3.69, SD = 0.98), CSM (*M* = 2.59, SD = 1.43), D (*M* = 2.18, SD = 1.03), and lower scores were observed for A (*M* = 1.30, SD = 0.93).

General psychological distress measured by the Symptom Checklist‐90‐Revised (SCL‐90‐R) was similarly elevated, with the mean GSI (GSI = 111.35, SD = 90.96) exceeding the clinical threshold (≥ 63). The highest subscale scores were observed in depression (*M* = 2.40, SD = 0.85), interpersonal sensitivity (*M* = 2.17, SD = 0.91), and obsessive‐compulsive symptoms (*M* = 1.93, SD = 0.85). Clinically relevant symptom levels were also evident for anxiety (*M* = 1.87, SD = 0.91), somatization (*M* = 1.35, SD = 0.83), and paranoid ideation (*M* = 1.48, SD = 0.82).

### 3.2 Autistic Traits, Sensory Sensitivities, and Social Cognition

The mean AQ‐10 score was 4.38 (SD = 1.51), with the clinical cut‐off score of ≥ 6. The mean GQ‐ASC total score was 50.41 (SD = 8.83), with the clinical cut‐off of ≥ 54. Based on these thresholds, 25.0% of the sample (*n* = 41) exceeded the cut‐off on the AQ‐10, whereas 42.7% (*n* = 70) exceeded the cut‐off on the GQ‐ASC. However, only 14.6% (*n* = 24) scored above the threshold on both measures. Sensory sensitivities revealed moderate sensory hyper‐responsivity, with a mean score of 15.22 (SD = 5.20) (lower scores reflecting greater hypersensitivity). Social cognition and theory of mind were assessed with the SET. Participants' mean scores were: cognitive empathy (CI: *M* = 4.62, SD = 1.20), emotional empathy (EA: *M* = 4.74, SD = 1.17), and IA (*M* = 4.90, SD = 1.24). Although overall SET performance was within normative ranges, subtle differences emerged across subgroups as detailed in later analyses.

### 3.3 Associations Among Neurodivergent Traits, ED Severity, and Social Cognition

Pearson correlations revealed a moderate positive association between AQ‐10 and GQ‐ASC total scores (*r* = 0.17, *p* = 0.025), supporting some shared variance between the two autistic trait measures. However, the AQ‐10 showed no significant correlations with sensory sensitivity (SPQ‐10) or any of the SET subscales, showing no significant correlations to variations in sensory processing and social cognition within this population.

Conversely, the GQ‐ASC total score demonstrated significant negative correlations with all three SET domains: CI (*r* = −0.16, *p* = 0.047), EA (*r* = −0.28, *p* < 0.001), and IA (*r* = −0.27, *p* = 0.001), as well as with the overall SET total score (*r* = −0.31, *p* < 0.001). Subscale analyses indicated that difficulties in socializing and restricted interests were most strongly related to lower social cognitive performance: socializing difficulties negatively correlated with CI (*r* = −0.18, *p* = 0.030), EA (*r* = −0.18, *p* = 0.030), and IA (*r* = −0.22, *p* = 0.010); restricted interests were similarly associated with CI (*r* = −0.17, *p* = 0.048) and EA (*r* = −0.22, p = 0.010).

**FIGURE 1:**
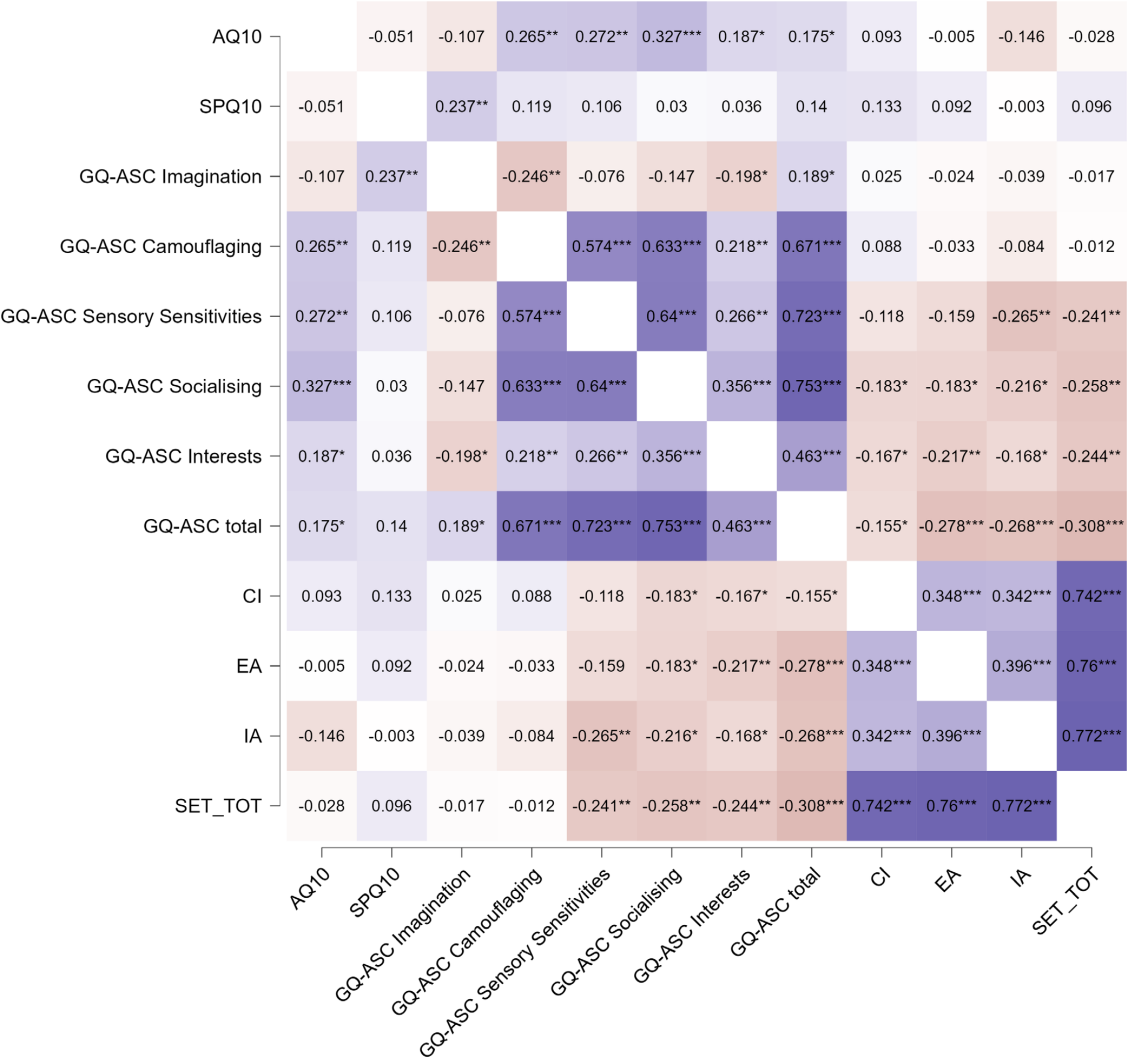
Heatmap of Pearson correlation coefficients between autistic traits (AQ‐10, GQ‐ASC total and subscales), sensory sensitivity (SPQ‐10), and social cognition (SET subscales: Cognitive empathy [CI], emotional empathy [EA], intention attribution [IA], and SET total). Purple indicates positive correlations, red indicates negative correlations. Darker shades represent stronger relationships. Notably, GQ‐ASC total and its subscales showed significant negative correlations with all SET subcomponents, while AQ‐10 demonstrated weaker or nonsignificant associations. *p < 0.05, **p < 0.01, ***p < 0.001.

No significant associations were found between camouflaging or sensory sensitivities and social cognition. However, both dimensions showed moderate positive correlations with the overall GQ‐ASC score. See Figure 1 for details.

### 3.4 Identification of Distinct Neurocognitive and Sensory Profiles

The hierarchical cluster analysis yielded a four‐cluster solution that identified distinct profiles across autistic traits, sensory sensitivity, social cognition, and ED severity. The four‐cluster solution was selected based on visual inspection of the dendrogram, supported by clinical interpretability (see Figure S1).

The analysis identified four distinct profiles: (1) a neurodivergent high‐risk group, (2) a sensory‐reactive but socially intact group, (3) a classically symptomatic but low‐risk profile, and (4) a cognitively compensated but clinically distressed subgroup.

Cluster 1 (*n* = 36) was characterized by high autistic traits (AQ‐10: *M* = 4.42, SD = 0.91; GQ‐ASC: *M* = 57.30, SD = 10.48) and high sensory sensitivity (SPQ‐10: *M* = 17.39, SD = 3.52), combined with low social cognition across all SET dimensions (CI: *M* = 3.50, SD = 1.16; EA: *M* = 3.72, SD = 1.39; IA: *M* = 3.36, SD = 1.36). Participants in this cluster exhibited clinical levels of ED severity (EDE‐Q: *M* = 4.46, SD = 1.00), marked body image disturbance (BUT‐TOT: *M* = 3.54, SD = 1.02), and substantial psychological distress (SCL‐90‐R: *M* = 154.82, SD = 46.16). Diagnostic distribution included 9 ANbp, 7 ANr, 8 BED, 8 BN, and 4 OSFED cases.

Cluster 2 (*n* = 42) presented with low autistic traits (AQ‐10: *M* = 3.31, SD = 0.87; GQ‐ASC: *M* = 45.24, SD = 10.35) and the highest social cognition scores (CI: *M* = 5.17, SD = 0.94; EA: *M* = 5.64, SD = 1.05; IA: *M* = 5.69, SD = 1.03), alongside higher levels than other clusters of sensory sensitivity (SPQ‐10: *M* = 17.67, SD = 3.80). Participants reported the lowest ED severity across clusters (EDE‐Q: *M* = 3.29, SD = 1.02), the lowest body image disturbance (BUT‐TOT: *M* = 2.57, SD = 0.95), and relatively low psychological symptoms (SCL‐90‐R: *M* = 100.10, SD = 36.37). Diagnostic distribution: 4 ANbp, 14 ANr, 12 BED, 6 BN, 6 OSFED.

Cluster 3 (*n* = 34) showed low autistic traits (AQ‐10: *M* = 3.18, SD = 0.92; GQ‐ASC: *M* = 44.10, SD = 10.40) and the lowest sensory sensitivity (SPQ‐10: *M* = 8.50, SD = 2.99), with moderate social cognition (CI: *M* = 4.21, SD = 1.09; EA: *M* = 4.59, SD = 1.17; IA: *M* = 4.94, SD = 1.09). This group exhibited clinical ED severity (EDE‐Q: *M* = 4.27, SD = 1.13), moderate body uneasiness symptoms (BUT‐TOT: *M* = 3.13, SD = 0.90), and the lowest overall psychological distress (SCL‐90‐R: *M* = 71.70, SD = 25.78). Diagnostic distribution: 4 ANbp, 14 ANr, 6 BED, 8 BN, 2 OSFED.

Cluster 4 (*n* = 52) had the highest auristic traits (AQ‐10: *M* = 5.37, SD = 1.18; GQ‐ASC: *M* = 53.93, SD = 10.62), along with preserved social cognition (I: *M* = 5.21, SD = 0.99; EA: *M* = 4.81, SD = 1.16; IA: *M* = 5.29, SD = 1.14). Despite moderate‐to‐high social cognitive performance, participants exhibited the highest ED severity (EDE‐Q: *M* = 4.92, SD = 0.94), increased sensory sensitivity (SPQ‐10: *M* = 16.13, SD = 4.00), marked body image disturbance (BUT‐TOT: *M* = 3.44, SD = 0.91), and elevated psychological distress (SCL‐90‐R: *M* = 116.28, SD = 35.39). Diagnostic distribution: 3 ANbp, 10 ANr, 9 BED, 20 BN, 10 OSFED.

**FIGURE 2:**
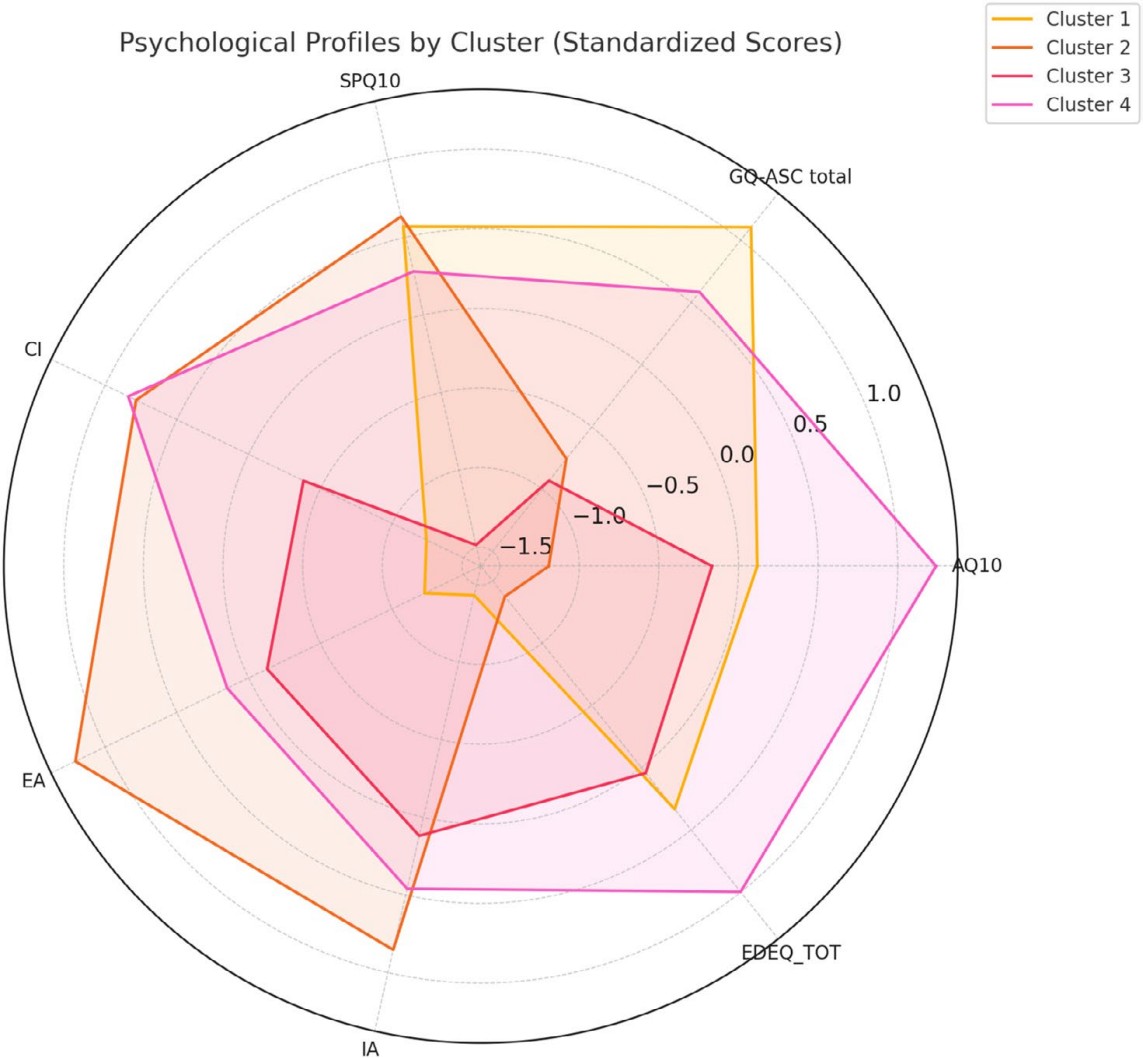
Radar plot displaying standardized mean scores across clustering variables for the four hierarchical clusters. Variables include autistic traits (AQ‐10, GQ‐ASC total), sensory sensitivity (SPQ‐10), social cognition (SET subscales: Cognitive empathy [CI], emotional empathy [EA], intention attribution [IA]), and eating disorder severity (EDE‐Q total). Scores are z‐transformed to allow for direct comparison across scales. Each line represents the average psychological profile of one cluster. The figure highlights distinct neurocognitive and clinical profiles, including a high‐risk neurodivergent group (Cluster 1), a sensory‐sensitive but cognitively intact group (Cluster 2), a low‐risk classically symptomatic profile (Cluster 3), and a cognitively compensated but clinically distressed subgroup (Cluster 4).

The standardized psychological profiles of the four clusters are illustrated in Figure 2.

To explore whether the distribution of ED diagnoses varied across the identified clusters, a chi‐squared test of independence was conducted. Results revealed a statistically significant difference in diagnostic composition among clusters, *χ* ^2^(12) = 22.08, *p* = 0.037, indicating that diagnostic categories were not evenly distributed across groups. However, post hoc pairwise comparisons applying Bonferroni correction did not yield statistically significant differences. These findings suggest that while diagnostic categories vary across clusters, the differences reflect complex clinical heterogeneity rather than strict categorical separation.

### 3.5 Comparisons Between Clusters

To confirm the validity of the identified cluster solution, both one‐way ANOVAs and non‐parametric Kruskal–Wallis tests were conducted for each clustering variable and additional clinical measures. Specifically, analyses included autistic traits (AQ‐10, GQ‐ASC total), sensory sensitivity (SPQ‐10), social cognition (SET subscales: CI, EA, IA), ED severity (EDE‐Q global score), body image disturbance (BUT‐TOT), and general psychopathology (SCL‐90‐R).

Statistical testing revealed significant differences across all variables (all *p* < 0.001); supporting the meaningfulness and robustness of the four‐cluster structure. Detailed test statistics and corresponding *p* values for each analysis are presented in Table 2.

**TABLE 2:** ANOVA and Kruskal–Wallis test results across clusters.

Variable	ANOVA *F*	ANOVA *p*	Kruskal–Wallis *H*	Kruskal–Wallis *p*	Post hoc significant comparisons
AQ10	19.71	< 0.001	44.60	< 0.001	C1 vs. C2 (*p* = 0.006) C1 vs. C4 (*p* = 0.027) C2 vs. C4 (*p* < 0.001) C3 vs. C4 (*p* = 0.001)
GQ‐ASC total	32.66	< 0.001	61.61	< 0.001	C1 vs. C2 (*p* < 0.001) C1 vs. C3 (*p* < 0.001) C2 vs. C4 (*p* < 0.001) C3 vs. C4 (*p* < 0.001)
SPQ10	44.32	< 0.001	67.27	< 0.001	C1 vs. C3 (*p* < 0.001) C2 vs. C3 (*p* < 0.001) C3 vs. C4 (*p* < 0.001)
CI	28.86	< 0.001	61.13	< 0.001	C1 vs. C2 (*p* < 0.001) C1 vs. C4 (*p* < 0.001) C2 vs. C3 (*p* = 0.001) C3 vs. C4 (*p* < 0.001)
EA	25.59	< 0.001	55.63	< 0.001	C1 vs. C2 (*p* < 0.001) C1 vs. C3 (*p* = 0.050) C1 vs. C4 (*p* = 0.001) C2 vs. C3 (*p* < 0.001) C2 vs. C4 (*p* < 0.001)
IA	49.05	< 0.001	74.29	< 0.001	C1 vs. C2 (*p* < 0.001) C1 vs. C3 (*p* < 0.001) C1 vs. C4 (*p* < 0.001) C2 vs. C3 (*p* = 0.002)
EDE‐Q total	19.07	< 0.001	45.78	< 0.001	C1 vs. C2 (*p* = 0.001) C2 vs. C3 (*p* = 0.008) C2 vs. C4 (*p* < 0.001) C3 vs. C4 (*p* = 0.017)
SPQ10	44.32	< 0.001	67.27	< 0.001	C1 vs. C3 (*p* < 0.001) C2 vs. C3 (*p* < 0.001) C3 vs. C4 (*p* < 0.001)
BUT total	11.56	< 0.001	21.67	< 0.001	C1 vs. C2 (*p* < 0.001) C2 vs. C4 (*p* < 0.001)
SCL90R total	5.60	= 0.001	18.56	< 0.001	C1 vs. C3 (*p* < 0.001)

*Note*: Post hoc for Kruskal–Wallis.

Abbreviations: AQ‐10, Autism Spectrum Quotient; BUT, Body Uneasiness Test; CI, cognitive empathy (Story‐based Empathy Task); EA, emotional empathy (Story‐based Empathy Task); EDE‐Q, Eating Disorder Examination Questionnaire (global score); GQ‐ASC, Girls Questionnaire for Autism Spectrum Condition; IA, intention attribution (Story‐based Empathy Task); SCL90R, Symptom Checklist‐90‐Revised; SPQ‐10, Sensory Perception Quotient.

We conducted additional analyses to explore whether age, duration of illness, and BMI varied across the identified clusters. A Kruskal–Wallis test revealed a statistically significant difference in age (*χ* ^2^(3) = 9.71, *p* = 0.021). Post hoc pairwise comparisons using Bonferroni correction indicated that this effect was primarily driven by a significant difference between Cluster 4 (*M* = 28.69, SD = 10.35) and Cluster 3 (*M* = 27.62, SD = 8.99; *p* = 0.012); while no other comparisons reached significance. Mean ages were also similar across Cluster 1 (*M* = 27.92, SD = 10.78) and Cluster 2 (*M* = 28.21, SD = 10.66), suggesting limited developmental variation between most subgroups.

In contrast, the duration of illness did not significantly differ between clusters (*H*(3) = 3.07, *p* = 0.380), with mean durations as follows: Cluster 1 (*M* = 7.72 years, SD = 7.18), Cluster 2 (*M* = 6.19 years, SD = 5.45), Cluster 3 (*M* = 8.74 years, SD = 6.65), and Cluster 4 (*M* = 6.06 years, SD = 5.84).

Regarding BMI, a Kruskal–Wallis test indicated no significant differences across clusters (*χ* ^2^(3) = 5.82, *p* = 0.116). Mean BMI values were: Cluster 1 (*M* = 22.35, SD = 4.95), Cluster 2 (*M* = 21.90, SD = 4.28), Cluster 3 (*M* = 21.83, SD = 3.56), and Cluster 4 (*M* = 23.99, SD = 5.66). These findings suggest that while a significant difference was observed for age—particularly between Clusters 3 and 4—differences in illness duration and BMI were not significant contributors to the identified profiles.

### 3.6 Clinical Predictors of Cluster Membership

To assess the predictive validity of clinical and neurodevelopmental variables in determining cluster membership, a multinomial logistic regression was performed, with Cluster 1 set as the reference category. Standardized scores from the AQ‐10, GQ‐ASC total, SPQ‐10, the SET total score, EDE‐Q global score, BUT‐TOT, and SCL‐90‐R were included as predictors. To enhance generalizability and reduce the risk of overfitting, a 5‐fold cross‐validation approach was employed.

The model showed excellent performance, with a macro‐average F1‐score of 0.93 and consistently high precision and recall across all four clusters. Specifically, F1‐scores were 0.96 for Cluster 1, 0.95 for Cluster 2, and 0.91 for both Cluster 3 and Cluster 4. The confusion matrix (Figure S2) demonstrated strong agreement between predicted and actual classifications, whereas the multiclass ROC curve (Figure S3) indicated robust discriminative capacity, with all area under the curve (AUC) values exceeding 0.90.

Full regression coefficients and significance values are reported in Table 3.

**TABLE 3:** Multinomial logistic regression predicting cluster membership (reference: Cluster 1).

Cluster comparison	Predictor	*B*	Exp (*B*)	*p*
Cluster 2 vs. Cluster 1	Intercept	−5.009		0.002
	AQ10	0.698	2.010	0.022
	SPQ10	0.198	1.219	0.411
	GQASC total	0.069	1.072	0.150
	SET total	−0.025	0.975	0.360
	EDEQ global	0.034	1.035	0.494
	BUT total	−0.007	0.993	0.854
	SCL90R total	0.036	1.037	0.497
Cluster 3 vs. Cluster 1	Intercept	−3.205		0.016
	AQ10	0.398	1.489	0.152
	SPQ10	0.362	1.437	0.061
	GQASC total	0.081	1.084	0.088
	SET total	−0.020	0.980	0.435
	EDEQ global	0.082	1.086	0.003
	BUT total	0.010	1.010	0.747
	SCL90R total	0.054	1.056	0.225
Cluster 4 vs. Cluster 1	Intercept	−6.397		< 0.001
	AQ10	0.712	2.039	0.012
	SPQ10	0.559	1.749	0.002
	GQASC total	0.092	1.097	0.039
	SET total	−0.007	0.993	0.797
	EDEQ global	0.070	1.073	0.014
	BUT total	0.024	1.024	0.325
	SCL90R total	0.058	1.060	0.236

*Note*: Table shows multinomial logistic regression coefficients predicting cluster membership (reference group: Cluster 1). Positive values indicate increased log‐odds of belonging to the corresponding cluster relative to Cluster 1. All predictors were standardized (*z*‐scores).

Abbreviations: AQ‐10, Autism Spectrum Quotient; BUT, Body Uneasiness Test; EDEQ, Eating Disorder Examination Questionnaire; GQ‐ASC, Girls Questionnaire for Autism Spectrum Condition; SCL90R, Symptom Checklist‐90‐Revised; SET, Story‐based Empathy Task; SPQ‐10, Sensory Perception Quotient.

## 4 Discussion

This study provides novel evidence for the existence of distinct neurodevelopmental profiles within a clinically diagnosed ED population. Using a dimensional, person‐centered approach, we identified four psychologically and cognitively meaningful clusters, advancing prior literature by integrating gender‐sensitive autism screening, sensory sensitivity, and theory of mind performance into a unified clinical framework.

Although autistic traits were assessed dimensionally across the entire sample, the majority of participants did not exceed the clinical cut‐off on either the AQ‐10 or the GQ‐ASC, suggesting that overt neurodevelopmental profiles are not representative of the broader ED population. However, the emergence of two distinct clusters (Clusters 1 and 4) characterized by elevated autistic traits and/or sensory sensitivities underscores the clinical relevance of identifying neurodivergent subgroups. These findings highlight the importance of moving beyond group‐level means to detect meaningful individual differences that may inform personalized interventions.

The emergence of a neurodivergent high‐risk profile (Cluster 1) was particularly striking. Characterized by high autistic traits, pronounced sensory sensitivity, and impaired theory of mind, this cluster also exhibited the highest levels of ED severity, body image disturbance, and general psychological distress. These findings align with previous research linking autism spectrum features—particularly female‐typical traits such as social camouflaging and internalized anxiety—to more complex, chronic, and treatment‐resistant ED presentations (Califano et al. 2024; Kinnaird, Norton, Stewart, and Tchanturia 2019; Westwood and Tchanturia 2017). The convergence of sensory hypersensitivity and social cognitive impairments may exacerbate aversive eating experiences, reduce emotional awareness, and hinder therapeutic engagement (Brede et al. 2020; Kerr‐Gaffney, Mason, et al. 2020).

In contrast, Cluster 2 combined low autistic traits with high social cognition and moderate‐to‐high sensory sensitivity. Despite some sensory reactivity, these individuals showed the lowest levels of ED severity and general psychopathology, suggesting a more resilient profile. This resilience may be attributable to a combination of protective psychological factors. Specifically, lower levels of autistic traits are associated with greater cognitive flexibility, improved emotion regulation, and fewer interpersonal challenges—all of which may reduce vulnerability to disordered eating behaviors (Kinnaird et al. 2020; Westwood et al. 2016, 2017; Westwood and Tchanturia 2017). High theory of mind and empathic functioning may further contribute by fostering stronger interpersonal adaptation and reducing feelings of isolation, thereby buffering against symptom escalation. Importantly, although this cluster showed the lowest clinical severity, the mean EDE‐Q score (*M* = 3.29, SD = 1.02) still exceeded normative values reported in Italian community samples (*M* = 1.6, SD = 1.2; Calugi et al. 2017), indicating clinically relevant pathology. Nonetheless, the combination of preserved social cognition and lower neurodevelopmental burden may facilitate emotional attunement and more adaptive coping strategies, supporting treatment engagement and overall psychological functioning (Leppanen et al. 2022; McClure et al. 2022; Monell et al. 2018). This dual role may help explain the comparatively lower clinical severity observed in individuals with high social cognition, such as those in Cluster 2.

Cluster 3, characterized by low autistic traits and minimal sensory sensitivity, showed moderate clinical severity, with relatively low psychological distress and body uneasiness symptoms. This profile corresponds to more “typical” ED presentations, particularly restrictive forms without notable neurodevelopmental comorbidity (Treasure and Schmidt 2013). Given their lower psychopathological burden and younger average age compared to Cluster 4, individuals in this group may represent earlier‐stage cases who are more aligned with clinical profiles typically observed in standard ED treatment settings. Clusters 2 and 3 offer an instructive comparison in understanding the protective role of social cognition. Both clusters were characterized by low autistic traits, yet differed in their cognitive‐emotional configurations. Cluster 2 demonstrated high social cognition and high scores of sensory sensitivity, whereas Cluster 3 showed lower sensory sensitivity and moderate social cognition. Despite its heightened sensory reactivity, Cluster 2 presented the lowest ED severity and psychological distress in the sample. This suggests that high social cognitive functioning may buffer the emotional and clinical impact of sensory hypersensitivity, supporting emotional attunement and adaptive interpersonal regulation. Conversely, Cluster 3—despite being less reactive to sensory input—demonstrated greater clinical severity than Cluster 2, possibly reflecting the absence of such a cognitive‐emotional buffer. These findings underscore the importance of interplay, rather than isolated traits, in shaping clinical outcomes.

Cluster 4 exhibited high levels of autistic traits alongside preserved social cognition, elevated ED severity, significant body image disturbance, and marked psychological distress. This configuration suggests a cognitively compensated or “masked” neurodivergent profile, characterized by individuals who—despite internal neurodevelopmental vulnerabilities—maintain an outward appearance of social competence. Cognitive compensation refers to the development of deliberate strategies to navigate social contexts, such as mimicking social behaviors, relying on intellectualized reasoning rather than intuitive social processing, or pre‐planning interpersonal interactions (Cook et al. 2021; Livingston et al. 2019). Masking, often discussed in the autism literature, involves the suppression of autistic traits in order to meet neurotypical expectations and is particularly prevalent among females and gender‐diverse individuals (Dean et al. 2017; Hull et al. 2020). While such strategies can foster superficial adaptation, they often come at a high psychological cost, including chronic stress, identity confusion, emotional exhaustion, and elevated risk for anxiety, depression, and self‐harm (Cage and Troxell‐Whitman 2019). In this cluster, the co‐occurrence of elevated autistic traits with relatively intact performance on theory of mind tasks may reflect this compensatory functioning. These individuals may perform well on structured social cognition assessments while still struggling with real‐life emotional reciprocity and embodied intersubjectivity, leading to underrecognition in both clinical and research settings. Importantly, such profiles are often missed by conventional autism screening tools, which tend to emphasize externally observable social deficits rather than internal experiences or adaptive effort (Allely 2019; Bargiela et al. 2016). The substantial psychological burden observed in this cluster—despite preserved cognitive skills—underscores the need for gender‐sensitive and developmentally informed assessments that can detect subtle or atypical presentations of neurodivergence, particularly in populations with complex psychiatric comorbidities such as EDs.

We observed a statistically significant age difference between clusters, with Cluster 4 being significantly older than Cluster 3. This finding may reflect distinct developmental trajectories in individuals with elevated autistic traits and preserved social cognition. In particular, it is possible that age is associated with a more established cognitive compensation style, shaped by accumulated social experience and adaptive mechanisms developed over time (Cook et al. 2021; Livingston et al. 2019). These patterns may not simply reflect illness chronicity—as duration of illness did not significantly differ between clusters—but may instead point to broader neurodevelopmental and maturational trajectories that shape the expression and management of neurodivergent traits across the lifespan. Prior research suggests that with age, individuals—particularly those with preserved social cognition—may develop increasingly refined compensatory or camouflaging strategies in response to social demands, which can obscure underlying difficulties and complicate both recognition and support (Cook et al. 2021; Dean et al. 2017; Hull et al. 2020). This age‐related variation highlights the importance of considering lifespan development in the study of neurodiversity and EDs, as age may modulate the way traits are integrated into psychological functioning without necessarily altering clinical chronicity.

Importantly, body image disturbance and general psychopathology significantly differed across all clusters. These variables tracked not only with ED severity but also with neurodevelopmental dimensions, particularly in the more distressed clusters (1 and 4). This supports prior evidence suggesting body image concerns as transdiagnostic markers of distress in both ED and autistic populations, potentially reflecting difficulties in embodiment, identity, and emotion regulation (Brede et al. 2020; Krumm et al. 2017; Longhurst 2023; Meneguzzo and Todisco 2024).

The observed differences in clinical severity highlight the importance of tailoring treatment intensity and focus. While some individuals may benefit from standard cognitive‐behavioral interventions, others—especially those with prominent neurodevelopmental features—may require adaptations addressing social cognition, rigidity, and sensory processing. Correlation findings suggest that the GQ‐ASC may offer complementary information to the AQ‐10, showing stronger associations with social cognitive performance in this sample. This pattern may indicate that the GQ‐ASC captures aspects of autism‐related traits—such as camouflaging or non‐stereotypical expressions—that are more common in female‐presenting individuals with EDs (Allely 2019; Tubío‐Fungueiriño et al. 2021). Moreover, sensory sensitivity emerged as a transdiagnostic vulnerability across profiles, aligning with models proposing its centrality in both EDs and neurodevelopmental conditions (van den Boogert et al. 2022).

### 4.1 Clinical Implications

The identification of distinct profiles supports a dimensional, person‐centered approach to ED care. Clinicians should be aware of the heterogeneity in autism‐related features among ED patients, particularly in those with severe or chronic courses. Routine screening for autistic traits, especially using tools sensitive to female and camouflaged expressions, may facilitate more accurate diagnosis and individualized interventions. Sensory sensitivities and theory of mind impairments should also be integrated into treatment planning, given their potential to influence engagement, emotional processing, and interpersonal dynamics in therapy.

Building on recent proposals to optimize treatment environments for trait sensitivity in EDs (Peterson et al. 2024), our findings support tailoring interventions according to neurocognitive profiles. For example, individuals with heightened sensory sensitivity may benefit from adapting the therapeutic setting (e.g., minimizing auditory, tactile, or olfactory stimuli), as well as graded exposure to distressing sensory experiences such as food textures, mirrors, or body‐focused cues (Petty et al. 2023; Valentine et al. 2020). Conversely, individuals with impaired social cognition may require explicit support with mentalization, perspective‐taking, or emotion recognition through targeted exercises (e.g., social role play, emotion labeling, or video‐based feedback). Even for those with preserved but effortful social functioning, such as individuals who camouflage, clinical environments may benefit from reducing interpersonal load and encourage authenticity over performance.

Notably, our findings suggest that the protective role of social cognition appears most salient in individuals with lower levels of autistic traits, such as those in Clusters 2 and 3. In these profiles, higher social cognitive functioning may mitigate emotional dysregulation and enhance interpersonal resilience, even in the presence of sensory sensitivity. In contrast, in clusters with high scores of autistic traits (Clusters 1 and 4), preserved social cognition—particularly in Cluster 4—does not appear sufficient to offset clinical severity. This may reflect the cumulative burden of internal overload, sensory reactivity, and cognitive compensation (e.g., camouflaging), which can mask functional difficulties while maintaining high distress (Cook et al. 2021; Livingston et al. 2019). These observations highlight the need to tailor interventions not only based on the presence of social cognition strengths, but also considering their broader neurodevelopmental context. Clinicians should remain attentive to cognitively compensated individuals who may present as socially skilled yet continue to struggle internally, requiring nuanced, affirming, and individualized approaches.

### 4.2 Limitations and Future Directions

Despite the strengths of a multidimensional, data‐driven approach, several limitations should be acknowledged. The sample included only cisgender females who were White and of Italian nationality, limiting the generalizability of the findings to male, gender‐diverse, and more ethnically or culturally diverse populations. The cross‐sectional design precludes CIs. The sample comprises clinically severe patients, with limited representation in terms of cultural diversity. Additionally, our sample did not include individuals with ARFID, as these patients are not routinely admitted to the inpatient unit from which our sample was drawn. Given the established link between ARFID and autism in younger cohorts, future research should explore how autistic traits manifest across a broader range of ED presentations, including ARFID. Moreover, no external validation—such as clinician‐rated autism diagnoses or longitudinal outcome data—was available to confirm the functional impact of cluster membership. While the logistic regression model demonstrated strong classification performance using cross‐validation, results should be interpreted with caution, as all analyses were conducted within a single‐site, female‐only sample. Due to the uneven distribution of ED diagnoses in our sample, we were not able to perform statistically robust analyses comparing diagnostic subtypes. Replication in larger, more diagnostically stratified samples and more diverse populations—ideally with external validation—will be important to confirm the clinical utility of the model and may help clarify how neurodivergent traits and sensory profiles vary across ED categories. Additionally, we were unable to collect systematic data on participants' prior or ongoing pharmacological treatments. Although all assessments were conducted at the beginning of inpatient care—prior to receiving psychological treatment within the unit—it is possible that prior medication use may have influenced certain psychological profiles. Finally, although the GQ‐ASC has been applied in adolescent samples, it was originally developed for adults, and more research is needed to confirm its clinical validity and utility in younger populations. These aspects should be considered when interpreting the findings.

While the EDE‐Q is a widely used tool for assessing ED psychopathology, prior research has noted limitations in its sensitivity for detecting certain binge behaviors, especially in individuals with BED (Reas et al. 2006). This may have influenced the assessment of symptom severity in this subgroup and should be considered when interpreting the results.

Although our sample included individuals across a wide age range, age was not used as a clustering variable. However, a significant age difference emerged between certain clusters, particularly between Cluster 3 and Cluster 4. This suggests that age‐related factors may partially contribute to the expression of neurodevelopmental features. Nonetheless, the clusters were primarily defined by psychological and cognitive dimensions, supporting the presence of meaningful profiles beyond simple age stratification. Future studies using age‐stratified analyses or longitudinal designs may help disentangle developmental effects and better capture trajectories of neurodivergent traits across the lifespan. Future research should include longitudinal designs, clinician‐rated autism assessments, and exploration of profile stability across time and treatment settings. Incorporating neurobiological or cognitive performance markers could further elucidate mechanisms underlying these subtypes. Future research should extend to gender‐diverse, male, and more ethnically heterogeneous populations to enhance the generalizability and applicability of findings.

## 5 Conclusion

This study sheds new light on the heterogeneity of EDs by identifying distinct neurodevelopmental and psychological profiles grounded in autistic traits, sensory sensitivities, and social cognition. Using a dimensional, person‐centered approach, we demonstrated that these factors meaningfully differentiate subgroups within the ED population, with clear implications for clinical severity and psychological distress. Notably, we identified both a high‐risk neurodivergent subgroup and cognitively compensated or sensory‐reactive profiles, which traditional diagnostic frameworks may overlook.

Beyond characterizing these subtypes, we showed that they can be predicted with high accuracy using clinical and cognitive measures, underscoring the translational potential of neurocognitive profiling. These insights advocate for a personalized, neurodevelopmentally informed framework in the assessment and treatment of EDs. By integrating autistic traits, sensory processing, and social cognition into conceptualizations of EDs, clinicians and researchers can move toward more inclusive, effective, and individualized care models.

## Author Contributions

**Paolo Meneguzzo:** conceptualization, investigation, writing – original draft, methodology, visualization, validation, software, formal analysis, data curation. **Marta Magno:** investigation, conceptualization, methodology, writing – original draft, writing – review and editing, data curation. **Alice Garolla:** investigation, writing – review and editing, data curation. **Elisa Bonello:** investigation, writing – original draft, writing – review and editing, data curation. **Elena Tenconi:** conceptualization, writing – original draft, writing – review and editing, validation, methodology, data curation, supervision. **Patrizia Todisco:** conceptualization, supervision, resources, funding acquisition, writing – review and editing, project administration.

## Ethics Statement

The authors assert that all procedures contributing to this work comply with the ethical standards of the relevant national and institutional committees on human experimentation and with the Helsinki Declaration of 1975, as revised in 2008. A specific approval of the research protocol was obtained by the Vicenza Ethics Committee (47/21).

## Consent

Written informed consent was obtained from all participants, or from parents or legal guardians if participants were underage, for their involvement in this study and inclusion in any published research paper.

## Conflicts of Interest

The authors declare no conflicts of interest.

## Supporting information


**Figure S1:** Hierarchical clustering dendrogram generated using Ward's method and Euclidean distance.
**Figure S2:** Confusion matrix of the multinomial logistic regression model evaluated using 5‐fold cross‐validation.
**Figure S3:** Multiclass receiver operating characteristic (ROC) curve for the multinomial logistic regression model using one‐vs‐rest comparisons.

## Data Availability

The data that support the findings of this study are available on request from the corresponding author. The data are not publicly available due to privacy or ethical restrictions.
