# Supplementary material for: The Hidden Spectrum Within Eating Disorders: Clustering Neurodivergent Traits and Sensory Sensitivity
Source: Int J Eat Disord. 2025 Aug 17;58(11):2167–81. doi: 10.1002/eat.24529 (PMC12605647; doi:10.1002/eat.24529)
Supplement: Supplementary file 1 — Figure S1: Hierarchical clustering dendrogram generated using Ward's method and Euclidean distance. Figure S2: Confusion matrix of the multinomial logistic regression model evaluated using 5‐fold cross‐validation. Figure S3: Multiclass receiver operating characteristic (ROC) curve for the multinomial logistic regression model using one‐vs‐rest comparisons. [file EAT-58-2167-s001.pdf]

The Hidden Spectrum Within Eating Disorders: Clustering Neurodivergent Traits and Sensory Sensitivity

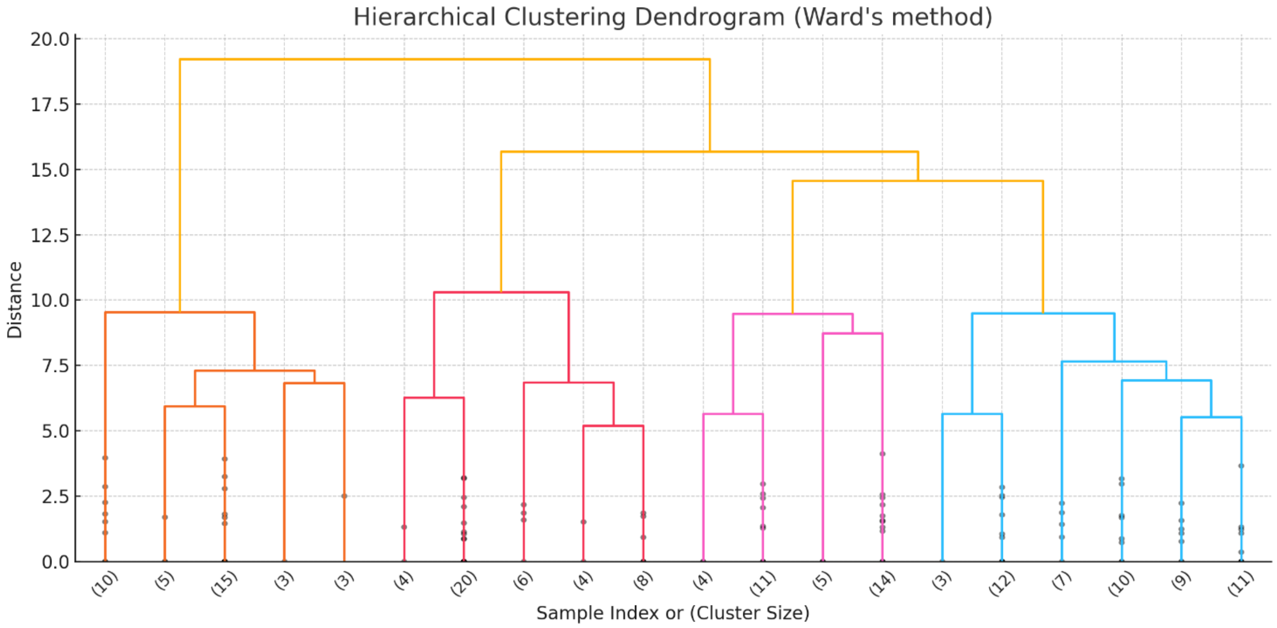

Supplementary Figure 1. Hierarchical clustering dendrogram generated using Ward's method and Euclidean distance. The figure displays the linkage distances across participants based on standardized values of autistic traits (AQ-10, GQ-ASC), sensory sensitivity (SPQ-10), social cognition (SET subscales: CI, EA, IA), and eating disorder severity (EDE-Q total). Visual inspection supported the selection of a four-cluster solution.

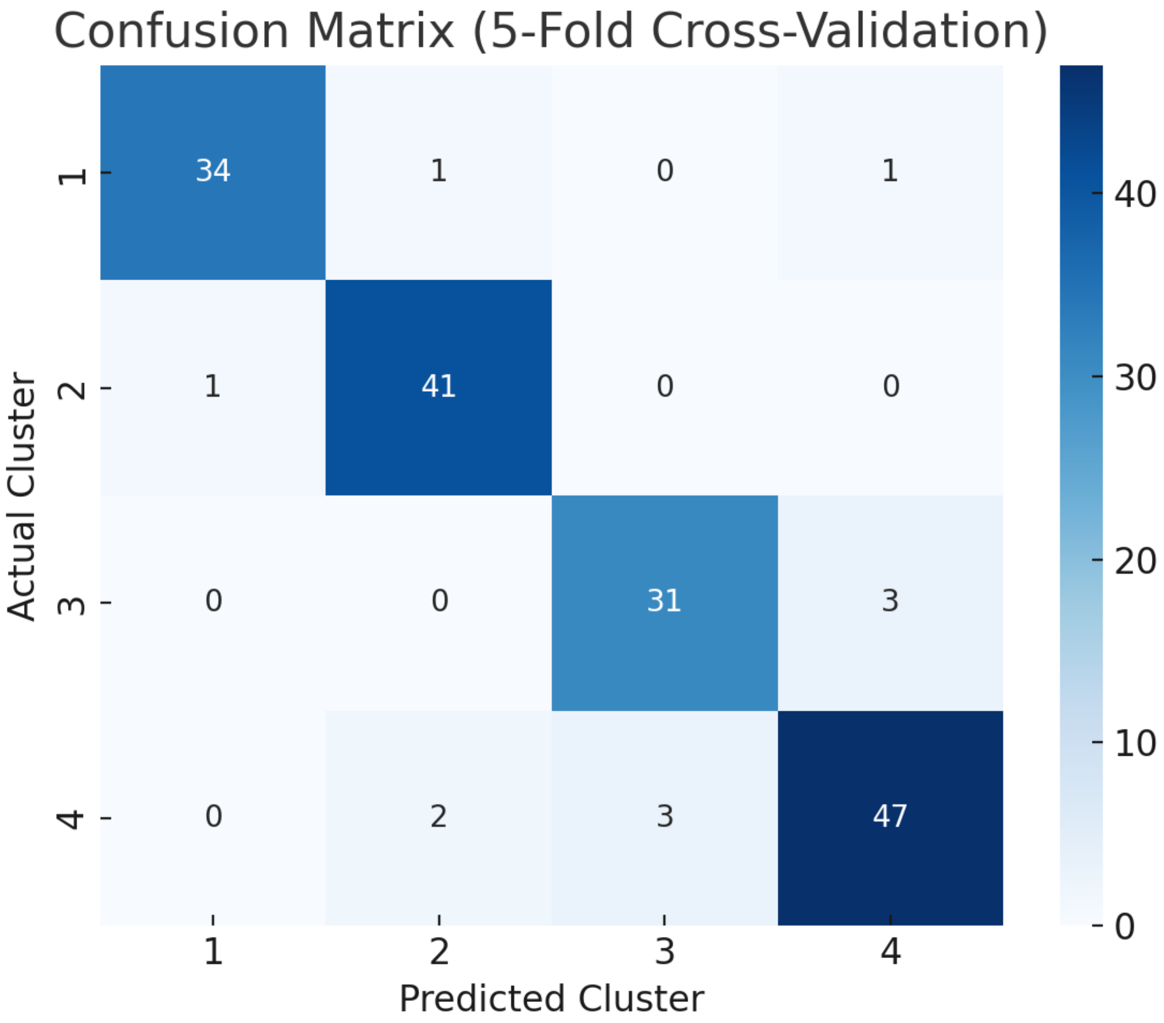

Supplementary Figure 2. Confusion matrix of the multinomial logistic regression model evaluated using 5-fold cross-validation. The model showed high classification accuracy across all four clusters, with the majority of predictions falling along the diagonal, indicating correct classifications. Rows represent actual cluster assignments; columns represent predicted cluster labels.

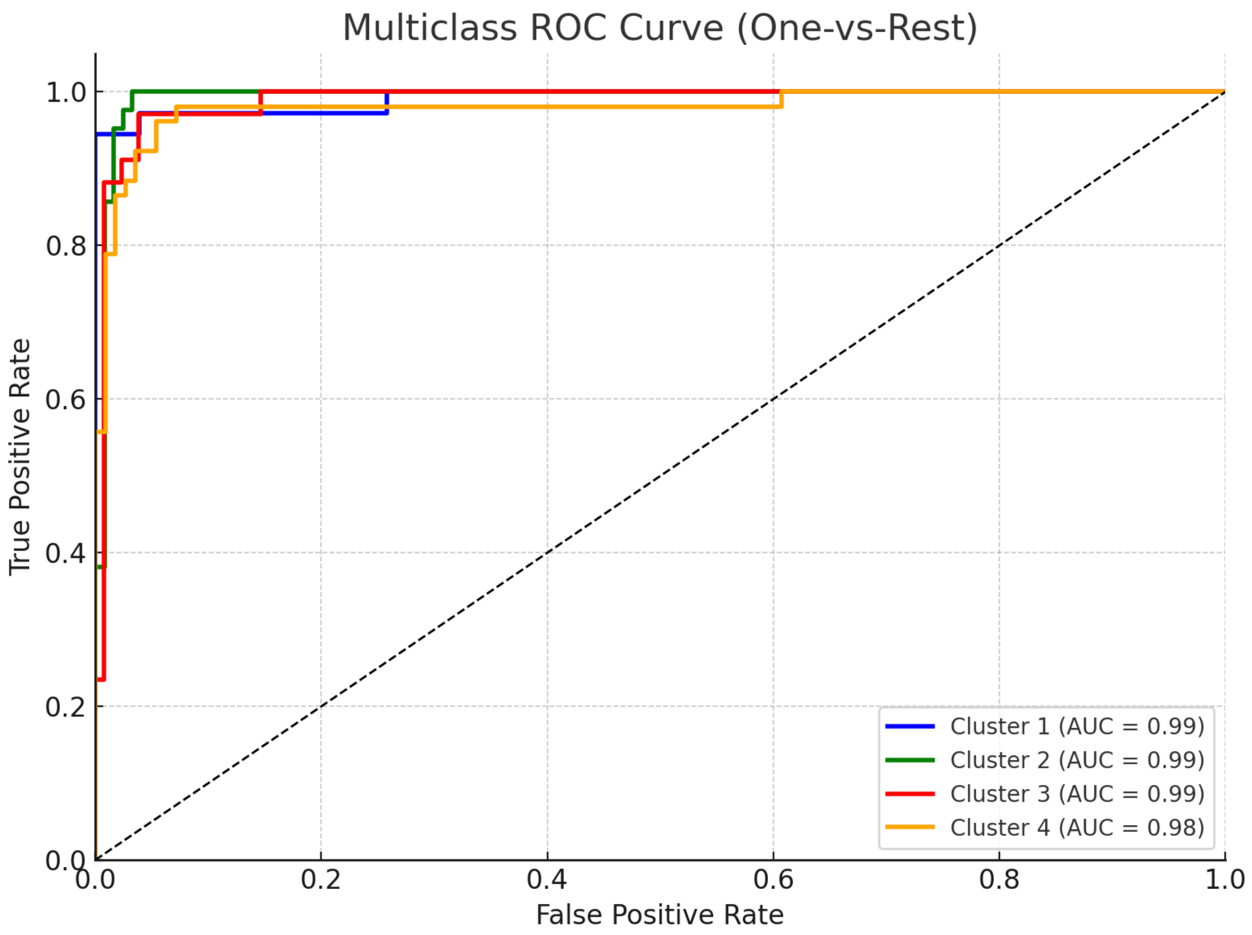

Supplementary Figure 3. Multiclass receiver operating characteristic (ROC) curve for the multinomial logistic regression model using one-vs-rest comparisons. The model demonstrated strong discriminative performance across all clusters, with area under the curve (AUC) values exceeding .90 for each class.
